# Endoscopic resection of gastric and esophageal cancer

**DOI:** 10.1093/gastro/gov050

**Published:** 2015-10-27

**Authors:** Bryan Balmadrid, Joo Ha Hwang

**Affiliations:** Division of Gastroenterology, University of Washington, Seattle, WA, USA

**Keywords:** endoscopic submucosal dissection, endoscopic mucosal resection, endoscopic ablation, early gastric cancer, early esophageal cancer

## Abstract

Endoscopic submucosal dissection (ESD) and endoscopic mucosal resection (EMR) techniques have reduced the need for surgery in early esophageal and gastric cancers and thus has lessened morbidity and mortality in these diseases. ESD is a relatively new technique in western countries and requires rigorous training to reproduce the proficiency of Asian countries, such as Korea and Japan, which have very high complete (*en bloc*) resection rates and low complication rates. EMR plays a valuable role in early esophageal cancers. ESD has shown better *en bloc* resection rates but it is easier to master and maintain proficiency in EMR; it also requires less procedural time. For early esophageal adenocarcinoma arising from Barrett’s, ESD and EMR techniques are usually combined with other ablative modalities, the most common being radiofrequency ablation because it has the largest dataset to prove its success. The EMR techniques have been used with some success in early gastric cancers but ESD is currently preferred for most of these lesions. ESD has the added advantage of resecting into the submucosa and thus allowing for endoscopic resection of more aggressive (deeper) early gastric cancer.

## Introduction

In the past, the treatment of gastrointestinal cancers centered on surgical resection. With steady advances in endoscopic techniques in the treatment of localized early cancers of the stomach and esophagus, more cancer patients are avoiding surgery altogether; in particular, developments in endoscopic mucosal resection (EMR) and endoscopic submucosal dissection (ESD) have resulted in fewer operations, leading to better patient tolerance, quality of life and overall cost savings. Although ESD is relatively new to western countries, Asian experience has shown very good rates of complete (*en bloc*) resection and low recurrence rates. Endoscopic devices and techniques have advanced to the point where full-thickness resection can be performed but, as deeper lesions have high risk for lymphatic invasion, endoscopic resections are typically limited to the mucosa and submucosa and are thus more appropriately treated with surgical resection with lymph node dissection.

### Endoscopic examination

With early esophageal and gastric cancers, the key component is a thorough examination of the lesion's surface characteristics (e.g. vascular and pit patterns) and an assessment for the depth of involvement. Firstly, a careful visual endoscopic examination is performed to determine the full extent of the lesion, since dysplastic extensions can be subtle. Newer ultra-high definition optics, along with narrow band imaging (NBI), near-focus visualization, image magnification, the use of a cap and use of chromoendoscopy with indigo carmine can all be used in combination to determine the appropriate resection field. As with any resection, obtaining dysplasia-free margins is the main objective. Electrocautery devices, such as a snare or needle knife, are used to mark a 2–5 mm clean outer margin.

### Endoscopic ultrasound evaluation

If deep invasion (in the submucosa or deeper) is suspected based on prior pathology or endoscopic evaluation, then endoscopic ultrasound examination should be performed, either with a radial array echoendoscope or an ultrasound catheter probe that fits through the working channel of a standard upper endoscope. It is important to determine the depth of invasion as involvement of the *muscularis propria* precludes endoscopic resection due to high risk of overt serosal perforation and very low likelihood of achieving an R0 resection, as nearly all of these will show lymphatic spread. Determining the depth of submucosal involvement can also be important, since this may also preclude EMR techniques.

### Endoscopic mucosal resection

**Figure 1.**
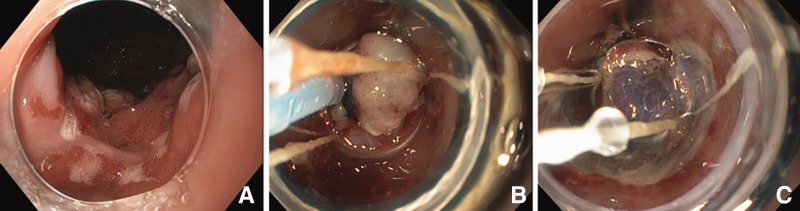
Ligation-assisted endoscopic mucosal resection (EMR) technique in Barrett’s esophagus. (**A**) Irregular areas of Barrett’s mucosa with clear margins are marked circumferentially with electrocautery. (**B**) Band ligation has been performed, creating a pseudopolyp, and now the snare has been placed above the band to perform electrocautery polypectomy. (**C**) Post-snare polypectomy with visualization of the submucosa.

In its simplest form, EMR has been used since 1955 [1, 2] and involves a submucosal injection/lift of the lesion to create a fluid cushion that creates a safety margin for cautery and cutting. Variations include a cap-assisted EMR, in which a plastic cap is attached to the end of the scope, allowing suction to bring a mucosal lesion into the cap; a snare is then positioned within the cap, ensnaring the base of the suctioned tissue, and electrocautery is applied to resect the tissue; the lesion can be removed whole or in piecemeal fashion. Ligation-assisted EMR is the most commonly used technique in the USA. A cap with single- or multiple-band ligators (similar to esophageal varices band ligators) is attached to the end of the scope. After application of suction to the lesion, small rubber bands are applied to the base of the suctioned tissue, creating a pseudopolyp that can be removed using basic polypectomy techniques (Figure 1).

EMR techniques can be successful in complete resections of lesions as large as 20 mm across [3], although lesions smaller than 10 mm typically allow the highest success for *en bloc* resection. It is common to perform a piecemeal resection of larger lesions but this does not allow for confirmation of complete resection by negative margins. Despite this, EMR still allows for diagnostic and prognostic information, even with incomplete resections. These samples allow for evaluation of lymphatic and blood vessels, which can predict lymph node metastasis.

Advantages of EMR include its relative simplicity, safety, and ability to obtain larger samples than biopsies. Limitations include a higher recurrence rate and lower rates of *en bloc* resection than ESD provides. Specifically, in larger lesions requiring multiple snare resections, cautery effects may obscure visualization. In general, EMR is less time-consuming than ESD [4, 5].

### Endoscopic submucosal dissection

**Figure 2.**
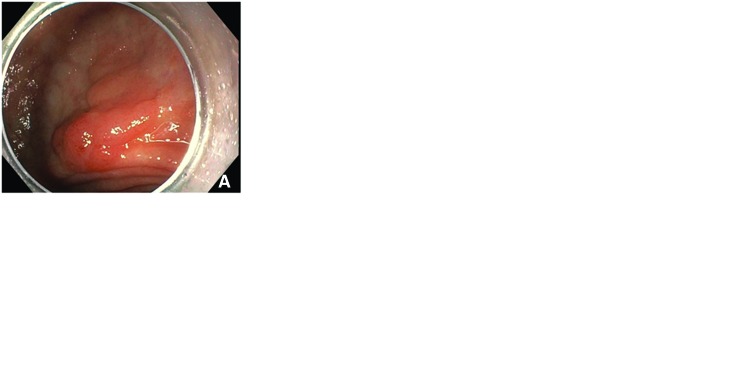
Endoscopic submucosal dissection (ESD) technique in early gastric cancer located at the incisura. (A) Mucosal lesion, spanning approximately 2 cm in white light view. (B) Mucosal lesion, giving cause for concern, in narrow band image view. (C) Perimeter of planned incision marked with electrocautery. (D) After circumferential incision. (E) After completion of dissection. (F) Resection specimen 34 mm x 29 mm.

The ESD technique arose from the high incidence of gastric cancer in Asian countries, particularly Japan and Korea. In order to reduce mortality from cancer, these countries established gastric cancer screening protocols for the general population. This led to an increase in the detection of early gastric cancers which, in turn, were amenable to endoscopic treatment. ESD was perfected in these countries and applied to different parts of the gastrointestinal (GI) tract, such as the esophagus. Adoption of ESD has been slow in western countries because of the steep learning curve in mastering this technique and the lack of volume due (i) to generally lower incidences of gastric cancer and (ii) lack of a screening program that may allow detection of early, endoscopically resectable cancers. ESD is a challenging technique that involves creating a large submucosal cushion through submucosal injections, and through the use of various cautery needle knife devices, cutting the lesion out in one piece (*en bloc*) (Figure 2). Extensive training and appropriate numbers of procedures are important in mastering this technique. Visualization and scope positioning can often be difficult and bleeding frequently occurs throughout the procedure. Complication rates and total endoscopy time will initially be high, but decrease with increased procedure volume and experience. In general, curative resection and recurrence rates are superior to conventional EMR [6–9].

## Early esophageal cancers

The incidence of esophageal cancer has been increasing worldwide [10, 11]. In the western world, esophageal adenocarcinoma (EAC) has become far more prevalent than squamous cell carcinoma (SCC) [12–14] while, in Asian countries, squamous cell carcinoma is very much in the majority, with adenocarcinoma accounting for only 4% of all esophageal malignancies [15]. Increased general use of endoscopy for abdominal symptoms and Barrett’s Esophagus surveillance protocols have led to the detection of early esophageal cancer that is amenable to endoscopic treatment.

### Barrett’s esophagus

Barrett’s esophagus (BE), defined as intestinal metaplasia of the esophageal squamous mucosa, is a precursor of EAC. Mostly a western disease, it is theorized that, with increased eradication of *Helicobacter pylori* and increased obesity, incidence of EAC may continue to rise in Asian countries [14]. Progression of BE to EAC is directly related to the presence of low-grade dysplasia (LGD) and high-grade dysplasia (HGD). The American Gastroenterology Association (AGA) technical review reports the progression of BE without dysplasia to cancer at 0.5% per year, while other studies suggest lower progression rates, with the Danish nationwide population-based study showing progression to carcinoma at 0.12% per year [16]. For LGD, carcinoma progression rates vary from 0.5% to 13.4% per year, while HGD is reported at 6% per year, based on an AGA technical review [17]. An interesting review of studies in which esophagectomies were performed on HGD showed that 12.7% had underlying submucosal invasive cancer [18], suggesting more aggressive evaluation of HGD that may involve EMR or ultrasound to determine depth of invasion. Based on a variety of national guidelines, HGD and intramucosal carcinoma (IMC) should preferably be treated with EMR, combined with ablation of any remaining Barrett’s mucosa [19, 20].

EMR or ESD should be performed on any raised or nodular lesions, which suggest advanced pathology. Once advanced lesions are resected and pathology reviewed, the remaining Barrett’s mucosa should be ablated with radiofrequency ablation (RFA), because of the risk of metachronous and recurrent lesions in remaining Barrett’s metaplasia. RFA has been shown to be safe and effective in ablating metaplasia and allowing for new squamous mucosa to take its place. It typically requires 2–4 sessions of ablation, in which either a 3 cm long balloon providing circumferential ablation (HALO 360, Medtronic, Sunnyvale, CA, USA) or an endoscope-mounted targeted probe (HALO 90, Medtronic, Sunnyvale, CA, USA) is used [21]. Success rates are well established, with United States Radiofrequency Ablation Registry of 857 patients, 84% of whom benefited from complete eradication of intestinal metaplasia with or without EMR [22], and in the UK National Halo RFA Registry of 335 patients, showing clearance of any dysplasia in between 81–86% of patients and complete eradication of intestinal metaplasia in 62% [23]. Complete eradication EMR, otherwise known as circumferential EMR, has been used with success but also with increased risk of stenosis [24–26]. EMR accompanied by RFA reduced the risk of cancer development, with high 5-year survival rates and 5-year intestinal metaplasia remission rates as high as 90% [14, 21, 27–35]. Recently, ESD combined with RFA has shown similar efficacy and safety [36]. RFA has been used on its own for the treatment of IMC with some success [37], but typical practice is to perform EMR on lesions that give cause for concern, especially if they are raised, as deeper lesions will not be appropriately treated with RFA, while endoscopic resection is important for staging.

### Squamous cell carcinoma

Esophageal EMR was first described in squamous cell carcinoma in 1991 [38, 39] and, in Asian countries, ESD is now the treatment modality of choice. A meta-analysis of eight Asian studies comparing ESD and EMR in the treatment of superficial esophageal cancer (primarily squamous cell carcinoma), demonstrated that ESD had a significantly higher *en bloc* resection rate (97.1% *vs*.** 49.3%; OR = 52.76; 95% CI 25.57–108.84) and a lower recurrence rate (0.3% *vs*.** 11.5%; OR = 0.08; 95% CI 0.03–0.23). Subset analysis showed no difference in the recurrence rate of lesions smaller than 2.0 cm (OR = 0.34; 95% CI 0.06–2.08). The procedure duration was significantly longer for ESD than for EMR [40].

### Depth of invasion in early esophageal cancer and risk of recurrence

The depth of invasion is directly related to lymph node metastasis and thus to rates of recurrence.; precise determination can be difficult at times. With only mucosal involvement (T1a), there is good response to endoscopic treatment. T1b lesions are more deeply invasive, involving the *muscularis* mucosa and the submucosa and thus carry a higher risk of recurrence. Another way of categorizing depth involves describing the deepest layer. Lesions confined the mucosa are labeled “m”. There are three levels of submucosal (sm) involvement referring to one-third involvement where sm1 tumors invade the superficial one-third of the submucosa and sm2 involves two-thirds and sm3 the lower one-third [41].

These categories predict the incidence of poorly differentiated carcinoma, lymphatic invasion, and venous invasion, all of which lead to incomplete resection or recurrence. Lymph node staging is important but sometimes difficult to characterize accurately without a formal surgical resection with lymph node harvesting. Classically, m1 (T1a) lesions are thought to rarely invade the lymphatic system (0.6%) [14]; however, a recent 2015 SEER database analysis of T1 lesions showed higher risk of invasion with T1a lesions, having lymph node metastasis prevalences of 6.4% and 6.9% for EAC and SCC, respectively. In addition, in a subgroup of patient who had undergone more extensive lymph node harvesting (>23), the incidences rose to 8.1% and 25%, respectively. In T1b (submucosal invasion) lesions, lymph nodes are involved in 19.6% and 20% for EAC and SCC, respectively. Lymph node metastasis is associated with worse 5-year survival, specifically in EAC but interestingly, this study did not show any significant effect on survival in SCC [42].

### Comparing ESD with EMR in early esophageal cancer

The goal for definitive treatment of early cancer is complete en bloc resection with clear tissue margins. Piecemeal resection, which is common in EMR cannot provide clear tissue margins and is associated with higher rate of recurrence. ESD technique leads to higher en bloc and curative resection rates compared to EMR. However, EMR is simpler to learn, easier to master and has shorter procedural duration compared to ESD [40]. EMR is simpler to learn, easier to master and has shorter procedural duration than ESD. When comparing the procedure durations of two different EMR techniques, a randomized trial for resection of Barrett’s-associated neoplasia demonstrated that ligation-assisted EMR was significantly faster than cap-assisted EMR, with median procedure times of 34 min *vs*.** 50 min, respectively (*P* = 0.02) with no differences in complication rates or quality of the resection specimens [43].

Japanese and Korean guidelines would recommend ESD for early esophageal carcinomas (EEC) but, in centers lacking ESD proficiency, EMR would be an appropriate alternative. Western countries are still acquiring proficiency in ESD [44, 45]. A 2012 German study reporting early experience with ESD showed a low complete *en bloc* resection rate of 38.5% in EAC [36], but a subsequent 2015 European report showed a figure of 83.9% for the same technique [46]. In Colombia, a recent review from one newly—but rigorously—trained endoscopist showed a high, tumor-free margin resection rate of 93% and median time of 61 minutes to resect tumors with a mean size of 19.8 mm [47]. To achieve high rates of cure and low complication rates in ESD, formalized, intensive training by observation, assisting, training with animal models, and by direct observation by a highly experienced endoscopist is key.

### Complications associated with endoscopic resection in early esophageal cancers

There is a statistically higher incidence of perforation in ESD than in EMR in EEC (OR = 2.19; 95%; CI 1.08–4.47; *P* = 0.03) and no statistical difference in bleeding rates between the two groups [40]. Formation of strictures is a matter of concern in ESD, EMR and RFA. EMR stricture rates are between 1% and 4.6% [48, 49], increasing slightly with a combination of EMR and RFA to 7.7% [49]. If EMR is used circumferentially, for example, to ablate residual Barrett’s esophagus, the stricture rate can be as high as 37% [50]. Strictures, based on meta-analysis, are more common in ESD than in EMR, occurring in 5–18% of cases [40, 51–56]; however, there appears to be a reducing trend after 2011, suggesting improved technique gained through more experience [54]. European studies suggest adopting empirical dilation within a week after ESD, along with continued weekly dilations with steroid injections to reduce the stenosis rate [7]. Dilation of esophageal, post-ESD strictures does carry cumulative risks, as patients can require more than 10 dilations and a per-patient perforation rate was reported to be as high as 4.1%, with a per-procedure rate of 0.37% [57].

### Other ablative therapies

Ablative therapies such as argon plasma coagulation (APC), photodynamic therapy (PDT), or cryotherapy are rarely used as monotherapy for early gastrointestinal cancers but, in small series, have shown to be a reasonable option for adjuvant therapy. Given the effectiveness of endoscopic resection, with or without RFA, these modalities have been relegated to salvage therapy.

APC is a non-contact method of thermal ablation, in which argon gas is ionized and used to conduct electrical current to the target tissue. The power settings are much higher than for typical use, with settings between 60–90 W at 1–2 L/min [58]. The data are mixed but show a reasonable remission rate for Barrett’s and HGD. With early EAC, there appeared to be a high recurrence rate; in a retrospective review from China, 6 out of 11 EACs recurred [59]. In early SCC there is better success, but these are again smaller numbers. In 19 patients with combined low-grade and high-grade esophageal squamous intraepithelial neoplasia and early SCC, 94.7% had a tumor eradication after 12 months of treatment with 22-month follow-up [60]. In another study of 17 patients treated with APC monotherapy for T1a & T1b SCC, there were 2 recurrences (9.5%), with a median follow-up of 36 months, requiring an average of 2 treatments and 15 minutes per treatment session [61].

PDT uses systemically infused porfimer sodium or 5-aminolaevulinic acid and causes significant photosensitivity; it is also relatively expensive. For these reasons, it has not had commercial success despite its potential efficacy. Sixteen out of seventeen patients with early EAC and underlying Barrett’s, who underwent PDT after endoscopic resection, were disease-free after median follow-up of 13 months [62]; however, a comparative study between RFA and PDT showed that RFA has better histological response and is more cost-effective, with less stricture formation [63]. Data are lacking for PDT in early esophageal squamous cell carcinoma and the technique cannot be recommended at this time.

Cryotherapy involves spray injection of liquid nitrogen and has been used mainly as salvage therapy when other modalities have failed. It is relatively safe, although perforations can occur due to the pressurized gas insufflation. A retrospective study of 79 patients with any T-staging, and who were not candidates for conventional therapy, showed 61.2–75% tumor eradication rate within a 10.6 month follow-up [64].

## Early gastric cancer

Gastric cancer is the most common form of malignant tumor in eastern Asia, eastern Europe and parts of Latin America. Overall, it is the fourth most common cancer and the second most common cause of cancer-related death worldwide [65]. Endoscopic surveillance is performed in many countries in the Asia-Pacific region, leading to detection of early gastric cancers, which are defined as lesions confined to the mucosa and submucosa, and are candidates for endoscopic resection. The Korean experience is a successful example of surveillance, where the proportion of detected early gastric cancers rose from 33% to 50% between 1999 and 2004, while advanced-stage gastric cancer decreased [66, 67].

### ESD in early gastric cancer

The classic indication for endoscopic resection of early gastric cancer involves differentiated adenocarcinoma confined to the mucosa and ≤2 cm when elevated and ≤1 cm if depressed. The expanded indication includes differentiated mucosal cancers of any size, differentiated submucosal cancers with less than 500 µm depth of invasion into the submucosa, and ulcerated differentiated cancers ≤3 cm. Beyond the expanded indication push the boundaries further and include larger differentiated intramucosal cancers >3 cm; differentiated submucosal cancers with less than 500 µm depth of invasion >3 cm; differentiated submucosal cancers with deeper invasion >500 µm, but ≤3 cm; non-ulcerated undifferentiated intramucosal cancers >2 cm (Table 1). As might be expected, complete resection rates drop dramatically as they approaches the limits of the expanded indication category, at 96.4%, 78.7% and 41.2% for classic, expanded, and beyond expanded indication groups, respectively [68–70]. Overall, *en bloc* resection rates are excellent. Korea has *en bloc* resection and complete *en bloc* resection rates of 95.3% and 87.7%, respectively, which has made ESD the preferred method of endoscopic resection [71]. Japan has similar outcomes, with *en bloc* resection rates of 92.7–96.1% and tumor-free margins in 82.6–94.5%, leading to curative resection rates of 73.6–85.4% [72]. Overall, early gastric cancer has a 90% 5-year survival rate, based on early studies [73, 74].

**Table 1.** Endoscopic submucosal dissection (ESD) indications for early gastric cancer

Classic indications	Expanded indications	Beyond expanded criteria
**Differentiated**	**Differentiated**	**Differentiated**
1. All lesions confined to mucosa and:	1. All nonulcerated lesions confined to mucosa	1. Ulcerated lesions confined to mucosa >3cm
A. Elevated ≤2 cm	2. Ulcerated lesions confined to mucosa and lesion ≤3cm	2. Submucosal involvement ≤500 um in depth and lesion >3cm
or B. Depressed ≤1 cm	3. Submucosal involvement ≤500 um in depth and lesion ≤3cm	3. Submucosal involvement >500 um in depth and lesion ≤3cm
**Undifferentiated**	**Undifferentiated**	**Undifferentiated**
N/A	4. Non-ulcerated lesion confined to mucosa and ≤2 cm	4. Non-ulcerated lesions confined to mucosa and >2 cm

Aside from its use in more aggressive early gastric cancers, ESD has also been studied for application to less aggressive lesions, gastric adenomas. Gastric adenocarcinoma arises via the ‘Correa cascade’ sequence of progression from inflammation to metaplasia to dysplasia to carcinoma [75]. Removing low-grade dysplasia or gastric adenomas would, in theory, interrupt this sequence. In western countries, the prevalence of adenomas is 0.5–3.75%, compared with 9–20% in Asian countries [76–78]. Concerning LGD and carcinoma without invasion, there are differences in semantics between Japan and western countries [79]. To reach a consensus, the World Health Organization (WHO) uses the terms ‘non-invasive low-grade-’ and ‘high-grade’ intraepithelial neoplasia and defines carcinoma as invading the *lamina propria* [80]. It is generally acceptable to perform ESD on high-grade lesions and carcinoma and to follow up with endoscopic surveillance for low-grade lesions; however, a case can be made that biopsies may under-stage lesions as low-grade dysplasia [81, 82], and the patient may thus be better served by undergoing ESD because of its proven efficacy and safety [79].

### Complications associated with ESD

Perforation rates are low, ranging from 1.2–5.2% [71, 83–85]. In western experience, perforation rates may be slightly higher but acceptable, with a range of 3.6–4.7% [47]. Delayed perforation has a smaller risk of about 0.5% [86, 87]. Factors increasing the risk of perforation include an associated ulcer, larger size and location of the lesion. The proximal and middle thirds of the stomach suffer higher perforation rates than the distal third, probably due to the thicker wall within the antrum and the need to sometimes perform resections in retroflexion with more proximal lesions [47, 88].

ESD is typically and frequently associated with immediate intraprocedural bleeding, which is nearly always controlled endoscopically. The amount of blood loss in immediate bleeding is sometimes difficult to quantify but a post-procedure, Day 1 drop in hemoglobin level of 2 g/dL is considered significant and occurs in 7% of cases [88]. Delayed bleeding is variably defined and ranges from 0–15.6% of patients undergoing ESD with larger lesions, longer procedure time and proximal lesions increasing the risk for delayed bleeding [88–90]. The proximal stomach has larger submucosal arteries that probably contribute to the higher risk of bleeding [90].

Stenosis rates are lower (0.7–1.9%) but are also highly dependent on the location, with lesions of the cardia and near the pylorus carrying higher risk rates of 17% and 7%, respectively [83]. Case reports of air embolism have led to the use of CO_2_ for most ESD procedures [91].

Considering these complications, ESD has been shown to be safe and effective in the elderly and in patients with chronic kidney disease, liver cirrhosis and other comorbid conditions [92, 93]. In western Countries, where surgical resection is the established treatment for early gastric cancer, it is probably these patients, who are not good candidates for surgery, who will undergo ESD.

### EMR in early gastric cancer

As described earlier, ligation-assisted EMR and cap-assisted EMR can be used for *en bloc* and piecemeal resection. It may not be the appropriate treatment modality for early gastric cancer with expanded and beyond expanded indications that involve the submucosa, as the lesion may not fully lift into the cap. Because of its ease of use and comparable efficacy and safety, it is still common, especially in western countries. *En bloc* resection and complete resection rates using this technique are typically lower at 51.7% and 42.2%, respectively, based on a recent meta-analysis [94]. There is a considerable difference in the durations of ESD procedures in expert hands, when compared with EMR. For large gastric lesions, the reported mean time to complete EMR is 25.8 ± 25.9 min, compared with (47.8 ± 38.3) – (84.0 ± 54.6) min for lesions removed by ESD [71, 95].

### Complications associated with EMR

Perforation rates are low at 0.8–2.9% [4, 94]. Intraprocedural bleeding was much lower in EMR than in ESD, at 7.6% [4] but, based on a meta-analysis, post-procedural bleeding rates remained the same for both EMR and ESD [94].

### Surveillance of early gastric cancer

Local recurrence rates after ESD appear to be low but metachronous recurrence appears to have a constant yearly rate of incidence; thus annual or biannual surveillance by Esophagogastroduodenoscopy (EGD) is recommended for at least 5 years following ESD [96].

## Conclusion

Endoscopic resection has been quite successful in treating early esophageal cancer and early gastric cancer, which are limited to the mucosa or superficial submucosa. ESD is the modality of choice for early GI cancers in Asian countries, given its high complete *en bloc* resection rates and low complication rates, even in the elderly and patients with significant comorbidities. More western gastroenterologists are being rigorously trained in this technique and gaining expertise. Limitations of ESD include the low volume of early gastric cancer in most western countries, and consistently large volumes are vital in gaining and maintaining proficiency in ESD. There are enough early esophageal cancers in western countries (specifically EAC), but it is hard to displace ligation-assisted EMR (with or without RFA) as the modality of choice, given the relative ease with which it can be mastered, shorter procedural time, good efficacy and low complication rates.

*Conflict of interest statement*: none declared.
